# Foveal Cone Structure in Patients With Blue Cone Monochromacy

**DOI:** 10.1167/iovs.63.11.23

**Published:** 2022-10-27

**Authors:** Emily J. Patterson, Angelos Kalitzeos, Thomas M. Kane, Navjit Singh, Joseph Kreis, Mark E. Pennesi, Alison J. Hardcastle, Jay Neitz, Maureen Neitz, Michel Michaelides, Joseph Carroll

**Affiliations:** 1 UCL Institute of Ophthalmology, University College London, London, United Kingdom; 2 Moorfields Eye Hospital NHS Foundation Trust, London, United Kingdom; 3 Cell Biology, Neurobiology & Anatomy, Medical College of Wisconsin, Milwaukee, Wisconsin, United States; 4 Casey Eye Institute, Oregon Health & Science University, Portland, Oregon, United States; 5 Ophthalmology, University of Washington, Seattle, Washington, United States; 6 Ophthalmology & Visual Sciences, Medical College of Wisconsin, Milwaukee, Wisconsin, United States

**Keywords:** blue cone monochromacy, S-cone, adaptive optics, opsin, wave guiding

## Abstract

**Purpose:**

Blue cone monochromacy (BCM) is a rare inherited cone disorder in which both long- (L-) and middle- (M-) wavelength sensitive cone classes are either impaired or nonfunctional. Assessing genotype-phenotype relationships in BCM can improve our understanding of retinal development in the absence of functional L- and M-cones. Here we examined foveal cone structure in patients with genetically-confirmed BCM, using adaptive optics scanning light ophthalmoscopy (AOSLO).

**Methods:**

Twenty-three male patients (aged 6–75 years) with genetically-confirmed BCM were recruited for high-resolution imaging. Eight patients had a deletion of the locus control region (LCR), and 15 had a missense mutation—Cys203Arg—affecting the first two genes in the opsin gene array. Foveal cone structure was assessed using confocal and non-confocal split-detection AOSLO across a 300 × 300 µm area, centered on the location of peak cell density.

**Results:**

Only one of eight patients with LCR deletions and 10 of 15 patients with Cys203Arg mutations had analyzable images. Mean total cone density for Cys203Arg patients was 16,664 ± 11,513 cones/mm^2^ (n = 10), which is, on average, around 40% of normal. Waveguiding cone density was 2073 ± 963 cones/mm^2^ (n = 9), which was consistent with published histological estimates of S-cone density in the normal eye. The one patient with an LCR deletion had a total cone density of 10,246 cones/mm^2^ and waveguiding density of 1535 cones/mm^2^.

**Conclusions:**

Our results show that BCM patients with LCR deletions and Cys203Arg mutations have a population of non-waveguiding photoreceptors, although the spectral identity and level of function remain unknown.

Normal human color vision is trichromatic, owing to the presence of three spectrally distinct types of cone. These are named long- (L-), middle- (M-), and short-wavelength sensitive (S-) based on the region of the visible spectrum to which they are maximally receptive. In humans, inherited color vision defects are quite common, although they vary widely in both type and severity. Red-green (protan and deutan) color vision defects are the most common, affecting nearly one in 12 men, and result from mutations in the *OPN1LW*/*OPN1MW* gene array, which encodes the L- and M-cone photopigments, respectively.^1^ A subset of these mutations results in Bornholm eye disease, which is typically characterized by high myopia in addition to a red-green defect.^2^^,^^3^ Yellow-blue (tritan) color vision defects involve mutations in the *OPN1SW* gene, which encodes the S-cone photopigment, although it only affects one in 500 individuals.^4^ Achromatopsia (ACHM) affects approximately one in 30,000 individuals and is associated with a loss of function of all three cone types, due most frequently to mutations in the genes encoding the α or β subunits of the cone cyclic nucleotide-gated ion channel (which is expressed in all cones, regardless of spectral subtype). Of growing interest is developing an improved understanding of high-resolution genotype-phenotype relationships in these various conditions.

Adaptive optics scanning light ophthalmoscopy (AOSLO) allows direct visualization of photoreceptor structure in the human retina in vivo.^5^ Confocal AOSLO collects light transmitted through a pinhole, and it is thought that visibility of a photoreceptor using this modality requires intact outer segment structure to facilitate waveguiding.^6^ In contrast, non-confocal split-detection AOSLO detects multiply-scattered light, which enables visualization of the cone inner segments regardless of their waveguiding properties.^6^ These AOSLO imaging modalities have revealed a wide range of cone mosaic phenotypes in individuals with red-green color vision defects, including completely normal topography,^7^ a contiguous mosaic of reduced density,^8^ loss of waveguiding with retained inner segment structure for a subset of cones,^9^ and even widespread loss of cone structure.^10^ These phenotypes generally correlate with the underlying genotype, although there have been discrepant findings in individuals with the same underlying genetic basis for their color vision defect.^11^^,^^12^ Similarly, diverse photoreceptor phenotypes are seen in patients with ACHM—generally intact cone structure in patients with *GNAT2* mutations,^13^ near absence of cone structure in patients with *ATF6* mutations,^14^ and absent cone waveguiding with remnant inner segment structure in patients with *CNGA3* or *CNGB3* mutations.^13^^,^^15^^,^^16^

The rarest inherited color vision defect is blue cone monochromacy (BCM), a condition in which both L- and M- cone classes are either impaired or non-functional.^17^ BCM is estimated to affect one in 100,000 men and is caused either by deletions involving the locus control region (LCR) upstream of the *OPN1LW/OPN1MW* gene array (one-step pathway) or missense mutations in the *OPN1LW/OPN1MW* genes (two-step pathway).^1^^,^^18^ The LCR is required for normal transcription of downstream genes within the array; its deletion therefore precludes expression of any downstream *OPN1LW/OPN1LW* genes.^17^ The most common missense mutation in BCM is Cys203Arg, which substitutes cysteine at position 203 with arginine, disrupting the disulphide bond with cysteine at position 126. This has been shown to alter protein folding, transport, and stability^19^ and is thought to lead to early degeneration of cone cells.^8^ AOSLO assessment of patients with BCM has thus far been limited to parafoveal confocal images,^20^ which restricts the ability to fully assess the extent of any remnant cone structure. In addition, the latter study only included those with the one-step genetic pathway. Here we used confocal and split-detection AOSLO imaging to further characterize foveal cone structure in individuals with BCM caused by both one-step and two-step genetic pathways.

## Methods

### Patients

Twenty-three male patients with genetically-confirmed BCM were recruited for high resolution imaging (Table 1; Supplementary Fig. S1). Eight patients had a deletion of the LCR and 15 had the Cys203Arg missense mutation expressed by the first two genes in the array (or the sole gene, when only one gene was present). Both genetic causes are expected to preclude expression of functional L and M opsin. This study followed the tenets of the Declaration of Helsinki and was approved by local institutional review boards (MCW: PRO17439 & PRO30741; UCL/Moorfields: 67979). Informed consent was obtained from all patients after the nature and possible consequences of the study were explained.

**Table 1.** Summary of the Genotype and Clinical Phenotype of Patients With Blue Cone Monochromacy

Family	Patient	Eye	Age (y)	Genotype	Axial Length (mm)	BCVA (LogMAR)	AOSLO Analysis
F1	JC_0078	OS	27	LCR deletion	28.7	0.84	No
F2	JC_0613	OD	18	LCR deletion	27.51	0.64	Yes
F3	KS_10992	OD	25	LCR deletion	25.83	0.80	No
F4	JC_11033	OS	53	LCR deletion	27.29	0.86	No
F5	JC_11230	OS	8	LCR deletion	24.24	0.70	No
F6: IV-3	JC_11237^†^	OD	6	LCR deletion	26.19	1.00	No
F6: II-1	JC_11239^†^	OS	75	LCR deletion	26.88	0.90	No
F6: III-8	JC_11266^†^	OD	35	LCR deletion	28.1	0.74	No
F7	JC_0183^*^	OD	24	M_C203R_	25.69	0.86	Yes
F7	JC_0184^*^	OS	21	M_C203R_	24.62	0.64	Yes
F8	MM_0187	OD	20	M_C203R_	26.36	0.64	Yes
F9	MM_0235	OD	18	M_C203R_	25.24	0.62	Yes
F10	JC_11532^*^	OS	49	M_C203R_	23.77	N/A	Yes
F10	JC_11585^*^	OS	54	M_C203R_	23.2	N/A	No
F11: IV-7	MP_10097^†^	OD	43	L_C203R_- M_C203R_	24.95	0.38	Yes
F11: V-2	MP_10116^†^	OD	10	L_C203R_- M_C203R_^‡^	27.17	0.92	No
F12	JC_10557^*^	OS	16	M_C203R_-M_C203R_	25.72	0.64	Yes
F12	JC_10558^*^	OS	19	M_C203R_-M_C203R_	25.51	0.54	Yes
F13	JC_10561	OS	50	M_C203R_-M_C203R_	25.8	0.62	No
F14	JC_11919	OD	20	M_C203R_-M_C203R_	28.03	0.66	No
F15: IV-1	JC_10066^†^	OS	24	L_C203R_-L_C203R_- M_C203R_-M	23.58	0.82	Yes
F15: IV-3	JC_10067^†^	OD	13	L_C203R_-L_C203R_- M_C203R_-M	22.81	0.68	No
F15: III-7	MP_10100^†^	OD	35	L_C203R_-L_C203R_- M_C203R_-M	26.82	0.72	Yes

C203R, Cys203Arg; BCVA, best corrected visual acuity; N/A, not available.

*The following are brothers: JC_0183 and JC_0184; JC_11532 and JC_11585; JC_10557 and JC_10558.

† Pedigrees shown in Supplemental Figure S1.

‡ Genotype inferred from MP_10097.

### Adaptive Optics Scanning Light Ophthalmoscopy

Before imaging, each eye imaged was dilated using one drop of phenylephrine hydrochloride (2.5%) and one drop of tropicamide (1%). Confocal and split-detection videos of the central photoreceptor mosaic were obtained with one of two previously described AOSLO systems, housed either at the Medical College of Wisconsin (MCW) or at Moorfields Eye Hospital (MEH).^6^^,^^13^ Because the functioning cones in this population (S-cones) are sparse and thought to be absent at the fovea,^21^ many patients might be expected to have eccentric fixation. To ensure that the AOSLO imaging protocol was centered on the anatomical fovea, the foveal reflex was located at the beginning of each imaging session by adjusting the depth of focus to the inner retinal layers. This location was used as the central anchor for mapping all other retinal locations for the imaging session. Raw videos were registered and averaged to produce images with a high signal-to-noise ratio, which were then montaged manually as previously described.^22^^,^^23^ Axial eye length was measured using the Zeiss IOL Master (Carl Zeiss, Meditec) and used to scale the AOSLO and other imaging modalities as previously described.^23^

### Aligning AOSLO and OCT Images

En face fundus images of the retina were acquired using either the Cirrus HD-OCT (Carl Zeiss Meditec, Jena, Germany), at MCW or Spectralis (Heidelberg Engineering, Heidelberg, Germany) at MEH. These images were manually inspected to ensure that the crosshair that marks the anatomical fovea did indeed coincide with the location of the foveal reflex. The lateral scale of each patient's fundus image was determined by dividing the nominal scan length by the assumed axial length (24.46 mm for the Cirrus; 24.835 mm for Spectralis) and multiplying by their measured axial length. Fundus images were then scaled to match that of the AOSLO montage, and the two images were aligned manually using anatomical landmarks (i.e., retinal vasculature) in Adobe Photoshop (Adobe Systems Inc., San Jose, CA, USA) (see Supplementary Fig. S2). For one patient (JC_10558), it was not possible to align the two modalities because of a lack of distinct landmarks in the fundus image.

The location of peak cell density (PCD) was identified from split-detection images by an experienced observer (E.J.P.), as previously described.^23^^,^^24^ Using the foveal crosshair from the fundus image, the distance between the anatomical fovea and location of PCD was measured (Table 2). It must be noted that a perfect correspondence between the anatomical fovea crosshair and location of PCD is not necessarily expected (even in the normal eye)^25^ because of factors such as warping, motion artifacts, lack of prominent anatomical landmarks at the fovea, and their variability in appearance across modalities. Despite this, the distance between the two locations did not exceed 186 µm in any of the patients for whom alignment was possible (Table 2; Supplementary Fig. S3) and was comparable to those observed for other foveal specializations in normal eyes.^25^^,^^26^ Given that alignment across modalities was not possible/reliable for all patients, but that the two locations aligned well overall, it was deemed appropriate to use the location of PCD as the “foveal center.”

**Table 2.** Summary of Cone Mosaic Metrics

		Confocal			Split-Detection		
Patient	PCD vs. F_a_ Distance (µm)	Density (cells/mm^2^)	PCD (cells/mm^2^)	N_c_ Across ROI (arcmin)	Density (cells/mm^2^)	PCD (cells/mm^2^)	N_c_ at PCD (arcmin)
JC_0613	54	1,535	4,698	2.80	10,246	22,103	1.29
JC_0183	107	1,154	3,668	3.17	6,024	12,930	1.69
JC_0184	101	1,121	5,482	2.59	6,767	11,560	1.78
MM_0187	60	2,241	6,824	2.32	9,908	19,685	1.37
MM_0235	1	3,409	8,167	2.12	12,568	52,490	0.84
JC_11532	73	728	6,140	2.45	9,346	27,289	1.16
MP_10097	29	3,073	7,701	2.19	12,784	45,997	0.89
JC_10557	186	2,296	5,777	2.52	40,207	60,928	0.78
JC_10558	N/A	1,657	5,831	2.51	14,954	22,934	1.27
JC_10066	91	2,978	6,483	2.38	20,787	34,530	1.03
MP_10100	105	N/A	N/A	N/A	33,292	48,920	0.87

F_a_, anatomical fovea; N/A, not analyzable.

Density was calculated using bound coordinates, whereas PCD and N_c_ were derived from unbound coordinates.

### Cone Density Analysis

A 300 × 300 µm region of interest (ROI) that was centered on the location of PCD (and also encompassed the anatomical fovea in all 10 patients for whom alignment was possible), was used for subsequent analysis. As the ROI comprised of a number of individual AOSLO images, the edges of these images were blended in Adobe Photoshop to produce a continuous ROI for analysis. Cone locations were marked using Mosaic Analytics (Translational Imaging Innovations Inc., Hickory, NC) which integrated a deep-learning based algorithm for identification of cones in split-detection images.^27^ All images were inspected twice by an experienced observer (EJP), who adjusted the coordinate locations and added/removed coordinates when necessary, to assess intra-observer repeatability.

**Figure 1.**
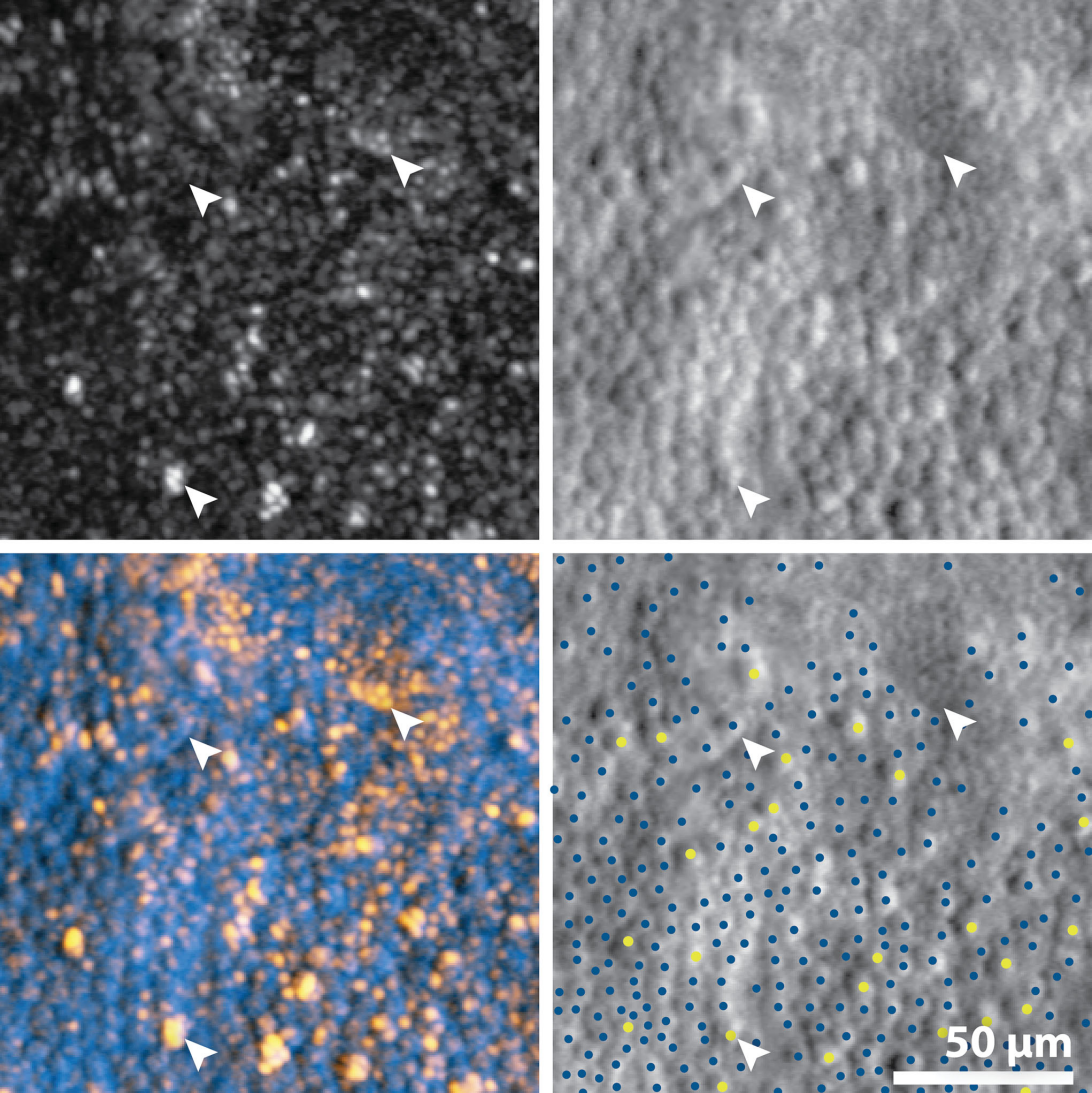
Adaptive optics scanning light ophthalmoscopy images from a patient with BCM (JC_10558), illustrating ambiguities in confocal images that could lead to misidentification of cones. Shown are the confocal (upper left), split-detection (upper right), false-colored composite (lower left), and marked split-detection images (lower right). In the false-colored composite, *yellow* represents the brightly reflective cones and rods visible in the confocal channel, and *blue* represents the inner segment structure shown in split-detection images. In the marked image, larger *yellow circles* denote waveguiding cones and smaller *blue circles* denote non-waveguiding cells, whose size is consistent with cones. *Bottom arrow*: a cone cell that has a multi-modal reflective appearance in the confocal image and could potentially be mistaken for a patch of rods. *Top left arrow*: there is no visible cone structure in the confocal image, but the corresponding split-detection image shows inner segment cone structure in this location. *Top right arrow*: a patch of rods. All images are taken from the top right quadrant of the 300 × 300 µm region of interest.

**Figure 2.**
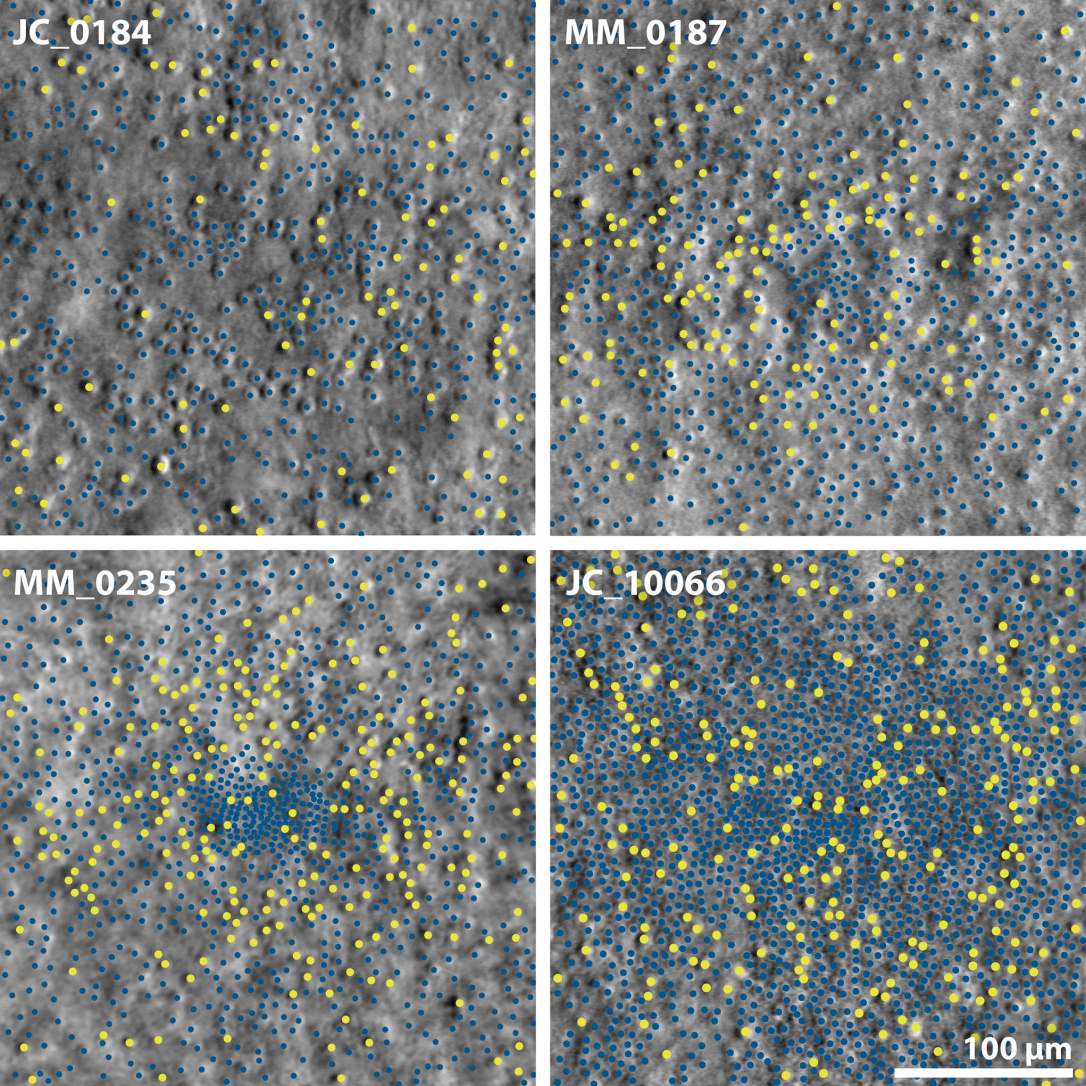
Split-detection images of four patients with blue cone monochromacy (centered on the location of peak cell density), demonstrating a range of retinal phenotypes. Waveguiding cones are marked with larger *yellow circles* and non-waveguiding cells, whose size is consistent with cones, are marked with smaller *blue circles*.

Quantifying waveguiding cones using confocal images poses a particular challenge in this population; the cones are often enlarged with a multimodal appearance, and hence can be mistaken for clusters of rods, which encroach further toward the fovea in these retinae (Fig. 1). Simply counting reflective structures in the confocal AOSLO image is therefore not a reliable measure of the number of waveguiding cones, and the same problems are encountered when using automated or image-processing methods. Therefore, waveguiding cones were estimated by manually identifying cones in a composite image (Fig. 2), which was created by summing the two image modalities (confocal and split-detection) using Fiji^28^—this enabled simultaneous visualization of reflectivity and the underlying inner segment structure, which helped to disambiguate multimodal cones from clusters of rods.

Further to assessing repeatability, one set of coordinates was inspected by two additional observers (J.C. and J.K.) to reach a consensus. This was achieved by overlaying the existing coordinates on the images in Mosaic Analytics and toggling between the split-detection and composite image to ensure correspondence between waveguiding cones marked on the composite image and cones marked on the split-detection image. All three observers were virtually present, using screen-sharing, for this process. These coordinates were used to calculate cone metrics (Table 2),^29^ as well as for subsequent analysis. PCD (Table 2) was calculated from unbound coordinates (i.e., including cells on the boundary of the analysis window, because of the sparsity of waveguiding cones) across a fixed window size of 37 µm using a custom Matlab script^30^ to enable comparison with previous literature.^25^^,^^31^ It is worth noting that, in the case of waveguiding cones, the location of PCD is not expected to coincide with the fovea, given the presence of the S-cone free zone, which has been shown to be present in patients with BCM^10^ and was also observed in many (but not necessarily all: see JC_10066 in Fig. 2) of these patients. Statistical analysis was completed on only one eye per patient using GraphPad Prism 9 (v. 9.4.1, GraphPad Software, San Diego, CA, USA).

To give an indication of the maximum theoretical resolution afforded by cones in the BCM retina, the Nyquist sampling limit of the cone mosaic (N_c_, arcminutes) was approximated using unbound cone density values (D, cones/mm^2^) at PCD (Table 2)^32^^,^^33^:

$$N_{c}=\left(\frac{60}{0.291}\right){\left(\frac{\surd 3}{2D}\right)}^{0.5}.$$

## Results

### AOSLO Imaging Success

Split-detection AOSLO images were analyzable in only one of eight patients with LCR deletions and 10 of 15 with Cys203Arg mutations. The failure to acquire analyzable data was due to severe nystagmus in only one of the five unanalyzable Cys203Arg and one of the seven LCR deletion patients, suggesting that nystagmus was not the limiting factor in analysis. One patient with an LCR deletion (JC_11239) had a cataract and one patient with Cys203Arg (JC_10561) had retinal dystrophy. The lack of success for the remaining sets was due to poor cooperation and/or low signal; the latter in some cases owing to either insufficient dilation or suppression of accommodation. Another factor that may have contributed to the low signal strength in many patients is axial length; the fact that axial length in the LCR deletion group was, on average, greater than that in the Cys203Arg group (27.39 and 25.30 mm, respectively, when discounting children under the age of 18 years) may explain at least some of the disparity in the success rate between groups.

### Repeatability

Of the 11 analyzable patients, the mean ± standard deviation (SD) total bound cone density (i.e., using only cells whose Voronoi region were fully contained within the ROI) across the central 300 µm × 300 µm ROI was 15,913 (±10,547) cones/mm^2^ for count 1 and 16,074 (±11,032) cones/mm^2^ for count 2. The total cone density values for both counts had a non-normal distribution (Shapiro-Wilk, *P* = 0.014 and 0.011) and so were log-transformed to assess intraclass correlation (ICC). The ICC coefficient for the two sets of log-transformed counts was 0.997, with a lower confidence interval (CI) of 0.990 and upper CI of 0.999 (*P* < 0.001), demonstrating excellent agreement. There was no statistically significant difference between the two sets of counts (Wilcoxon matched pairs, *P* = 0.898).

It was possible to further quantify waveguiding cones in 10 patients (Fig. 2; Supplementary Fig. S3)—the composite image from one Cys203Arg patient (MP_10100) could not be analyzed because of poor confocal image quality in the lower portion of the ROI. Of the 10 analyzable patients, the mean (±SD) bound density of waveguiding cones was 1458 (±1023) cones/mm^2^ for count 1 and 2000 (±977) cones/mm^2^ for count 2. The waveguiding cone density values for both counts had a normal distribution (Shapiro-Wilk, *P* = 0.076 and 0.276), so raw values were used to assess ICC. The ICC of both counts was 0.737, with a lower CI of 0.273 and upper CI of 0.927 (*P* = 0.003), demonstrating good agreement. However, a t-test revealed a statistically significant difference between the two sets of counts (*P* = 0.012), with a higher number of waveguiding cones being marked in the second attempt.

### Proportion of Waveguiding Cones

Given the comparably high variability of waveguiding cone density between the two intra-observer counts, the following analyses utilize the metrics obtained from the coordinates that were inspected by three observers to reach a consensus. The one patient with an LCR deletion (JC_0613) had a total bound cone density of 10,246 and waveguiding density of 1535 cones/mm^2^, yielding a proportion of 15% waveguiding cones. Mean (±SD) total cone density for Cys203Arg patients was 16,664 (±11,513, n = 10), and waveguiding density was 2073 (±963, n = 9) cones/mm^2^. Waveguiding cones thereby comprised 5% to 27% of the total density. Retinal stretching is unlikely to be a major source of the high variability, because there was no statistically significant correlation between axial length and either total (Spearman *r* = 0.261, *P* = 0.470, n = 10) or waveguiding cone density (Spearman *r* = 0.083, *P* > 0.843, n = 9) for Cys203Arg patients. Age-related degeneration is also unlikely to contribute significantly to the variability, as there was no significant correlation between age and either total (Spearman *r* = −0.255, *P* = 0.475, n = 10) or waveguiding cone density (Spearman *r* = −0.352, *P* = 0.352, n = 9). Finally, there was no significant correlation between best corrected visual acuity and either split-detection (total cone) N_c_ (Spearman *r* = 0.373, *P* = 0.325, n = 9), or waveguiding (presumably S-cone) N_c_ (Spearman *r* = 0.195, *P* = 0.650, n = 8).

## Discussion

Here we examined foveal cone structure in patients with genetically-confirmed BCM. Both genotypes studied are expected to result in a complete lack of functional L or M opsin; the difference being that for LCR deletions, the cone cell should never be exposed to opsin, whereas for Cys203Arg mutations, the cone cell is exposed to misfolded opsin. Total cone density within the central area of 300 × 300 µm (including both waveguiding and non-waveguiding cones) was, on average, around 40% of normal,^21^ suggestive of widespread failure to develop or early degeneration of cones. Because the waveguiding status of a cone is thought to provide an indication of its health and given that L/M cones are nonfunctional or absent in BCM, it would be reasonable to assume that any waveguiding cones are likely to be S-cones. Our estimates of S-cone density are therefore comparable to previous literature; however, histological estimates of S-cone density within the same sized region as analyzed here (calculated as the average of the density values measured at 0, 50, 100, and 150 µm by Curcio et al.; see their Fig. 8A)^21^ are lower (approximately 1030 cones/mm^2^ vs. our 2019 cones/mm^2^). Curcio et al.^21^ do acknowledge that their estimates “may be somewhat low” when compared to other histological studies, and shrinkage may also contribute to the disparity between estimates.

Crucially, the identity of the *non*-waveguiding cells (about 73%–94% of the total counted cells in our Cys203Arg patients) is not clear. There are at least three possibilities, which we explore in more detail below: (1) they represent non-waveguiding S-cones; (2) they represent enlarged non-waveguiding rods; or (3) they represent residual, nonfunctional L/M-cone inner segments.

### Non-Waveguiding S-Cone Hypothesis

The number of non-waveguiding cones was far greater than the number of waveguiding cones and also higher than that ordinarily expected of a normal S-cone population. This poses two queries for the S-cone hypothesis: why are they more numerous at the fovea than normal, and why are they not waveguiding? One explanation for their higher density within the foveal region could be that early induction of cone fate is altered, resulting in a greater number of cones being specified as S-cones than normal. This explanation is unsupported by evidence that S-cones develop earlier than L/M-cones^34^ and that opsin expression occurs later, with S-cone opsin expression preceding L/M opsin.^35^^–^^37^ An alternative explanation is that altered cone packing, rather than a greater overall number of S-cones, underlies the higher foveal density. If the L/M-cones failed to develop or degenerated before the fovea was fully formed, subsequent reorganization could lead to tighter S-cone packing within this space. This explanation is supported by AOSLO data in dichromats with Cys203Arg in only one of the *OPN1LW/OPN1MW* genes, which was suggestive of early cone death and subsequent reorganization of the remaining cones.^8^^–^^10^^,^^23^

To address the second query for the S-cone hypothesis (why do the majority not waveguide?), it is important to bear in mind that waveguiding status is not an unequivocal indication of cone health.^38^ Normal cone reflectance has been shown to vary over time,^39^ as well as in response to light stimulation.^40^ Moreover, measurable function has been found within lesions that lack visible cones in confocal AOSLO images,^41^ so it may be that the non-waveguiding cells observed in BCM are functional S-cones. Alternatively, the waveguiding query may be answered by the “bystander effect.” Secondary cone death occurs due to changes in oxygen levels in the outer retina and progressive oxidative damage to cones, which is observed in retinitis pigmentosa after rod death.^42^ Dying S-cones may temporarily retain their inner segment structure, but no longer waveguide, potentially accounting for the non-waveguiding cells in these patients. If this was the case, we would expect to observe a negative correlation between age and total cone density, which we did not. However, there is high variability in cone density in these patients so a larger sample across a larger age range, or longitudinal imaging of the same patients, may be needed to determine whether there is indeed any effect of age, and thereby assess feasibility of a theory involving secondary S-cone death. Overall, the hypothesis that the non-waveguiding cells represent an additional population of S-cones that are non-waveguiding seems unlikely but cannot be definitively ruled out, given the current data.

### Enlarged, Non-Waveguiding Rod Hypothesis

We observed numerous small waveguiding cells interspersed between the larger non-waveguiding cells, which were assumed to represent rods. It therefore also seems unlikely that these patients would have an additional population of rods that were enlarged and non-waveguiding. However, rods have been shown to be enlarged in other cone disorders such as ACHM,^6^ although they did waveguide. Little is known about the rod population in BCM (Patterson EJ, et al. IOVS 2021;62:ARVO E-Abstract 1879), but using AOSLO or dark-adapted microperimetry to assess rod structure and function across the retina would provide important data to properly evaluate this hypothesis.^43^

### Remnant, Nonfunctional L/M-Cone Hypothesis

The Cys203Arg mutation disrupts a highly conserved disulfide bond, which is essential for the correct folding, stability, and function of opsin. Cys203Arg opsin mutants do not fold correctly; they fail to bind 11-cis retinal and do not leave the endoplasmic reticulum, thereby producing protein overload that is toxic to the cells and subsequently causing death.^19^ AOSLO in patients with X-linked cone dysfunction caused by Cys203Arg in only one of the *OPN1LW/OPN1MW* genes, whereas the other remains unaffected, revealed lower cone density, with a mosaic that was consistent with early degeneration of affected cones, followed by reorganization of neighboring cones.^8^^–^^10^^,^^23^ In a recent study, using AO phase-sensitive optical coherence tomography, a patient with Cys203Arg encoded by *OPN1MW* showed no significant reduction in cone density from normal, which may be suggestive of retained M-cone structure. However, cone responses, measured using photostimulation-induced phase dynamics, showed no evidence of nonfunctional cones in this patient, as would have been expected if there was indeed any remnant M-cone structure.^12^ How might remnant L/M-cones survive in the BCM retina? A possible explanation for the current findings is that rods provide metabolic and structural support and/or electrical coupling, enabling survival of a small number of cones. In fact, it was recently shown that cones in *OPN1SW^−^^/^^−^* *OPN1MW* *^−^^/^^−^* mice maintain a normal dark current and continue to mediate visual signaling by relaying the rod signal through rod-cone gap junctions.^44^

If the non-waveguiding, cone-sized cells in these BCM patients did indeed represent remnant L/M-cones, *and* were amenable to treatments, such as gene replacement therapy, the theoretical maximum resolution (N_c_) that could be afforded (by total cones) would have the potential to improve from around 2 to 3 arcminutes to around 0.8 to 1.8 arcminutes. However, such an improvement would rely on successful treatment of *every single cone* at the foveal center; moreover, factors such as myopia,^45^ and reorganization at the postreceptoral (e.g., retinal ganglion cells)^32^ or postretinal level (e.g., visual cortex)^46^ are likely to limit the overall effect of treatment on functional vision. It is important to note that N_c_ represents the *theoretical* maximum resolution and does not account for the effects of the interocular optics, which are likely to reduce resolution,^32^^,^^47^ or fixational eye movements, which have the potential to increase resolution.^48^^,^^49^ Ultimately, it should be noted that cone presence is not synonymous with cone viability, and inferences about therapeutic potential in patients with BCM (based on the current data) should be approached with caution.

## Conclusions

Despite challenges surrounding interpretation of these photoreceptor images, the current study has revealed that BCM patients with LCR deletions and Cys203Arg mutations have a population of non-waveguiding cells—the size of which are consistent with cones—although the spectral identity and level of function remain unknown. Functional imaging studies using the optoretinogram could help determine whether these remnant cells are nonfunctional, as well as characterize their spectral identity (including rod vs. cone) if they are indeed functional.^50^^,^^51^ Finally, further longitudinal investigations could help to establish the stability of these cells, regardless of their identity.

## Supplementary Material

Supplement 1
