# Supplementary material for: Foveal Cone Structure in Patients With Blue Cone Monochromacy
Source: Invest Ophthalmol Vis Sci. 2022 Oct 27;63(11):23. doi: 10.1167/iovs.63.11.23 (PMC9624264; doi:10.1167/iovs.63.11.23)
Supplement: Supplement 1 [file iovs-63-11-23_s001.pdf]

Supplemental Figure 1

Pedigrees for Families 6, 11, and 15, as indicated in Table 1. Asterisks denote patients included in this study.

Family 6

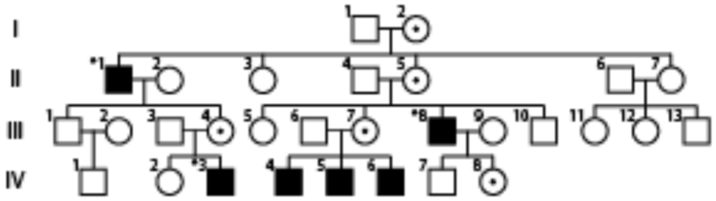

Family 11

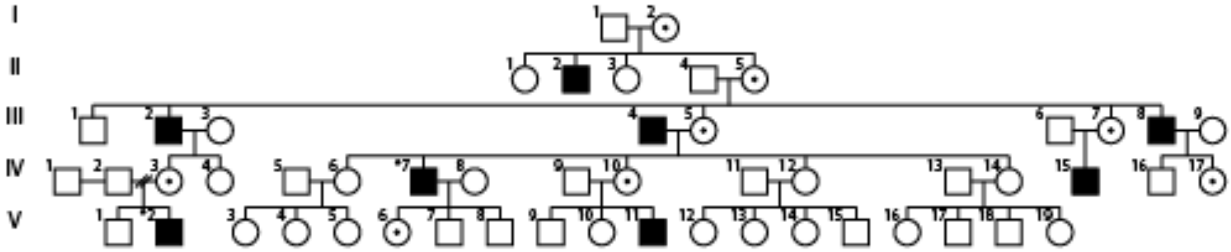

Family 15

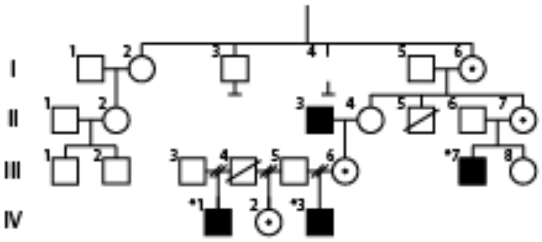

Supplemental Figure 2

Alignment of the confocal adaptive optics scanning light ophthalmoscopy montage and the fundus image (Cirrus HD-OCT, Carl Zeiss, Meditec) for JC_10557; the patient with the largest displacement (186 μm) between the locations of peak cell density (white square) and anatomical fovea (white circle).

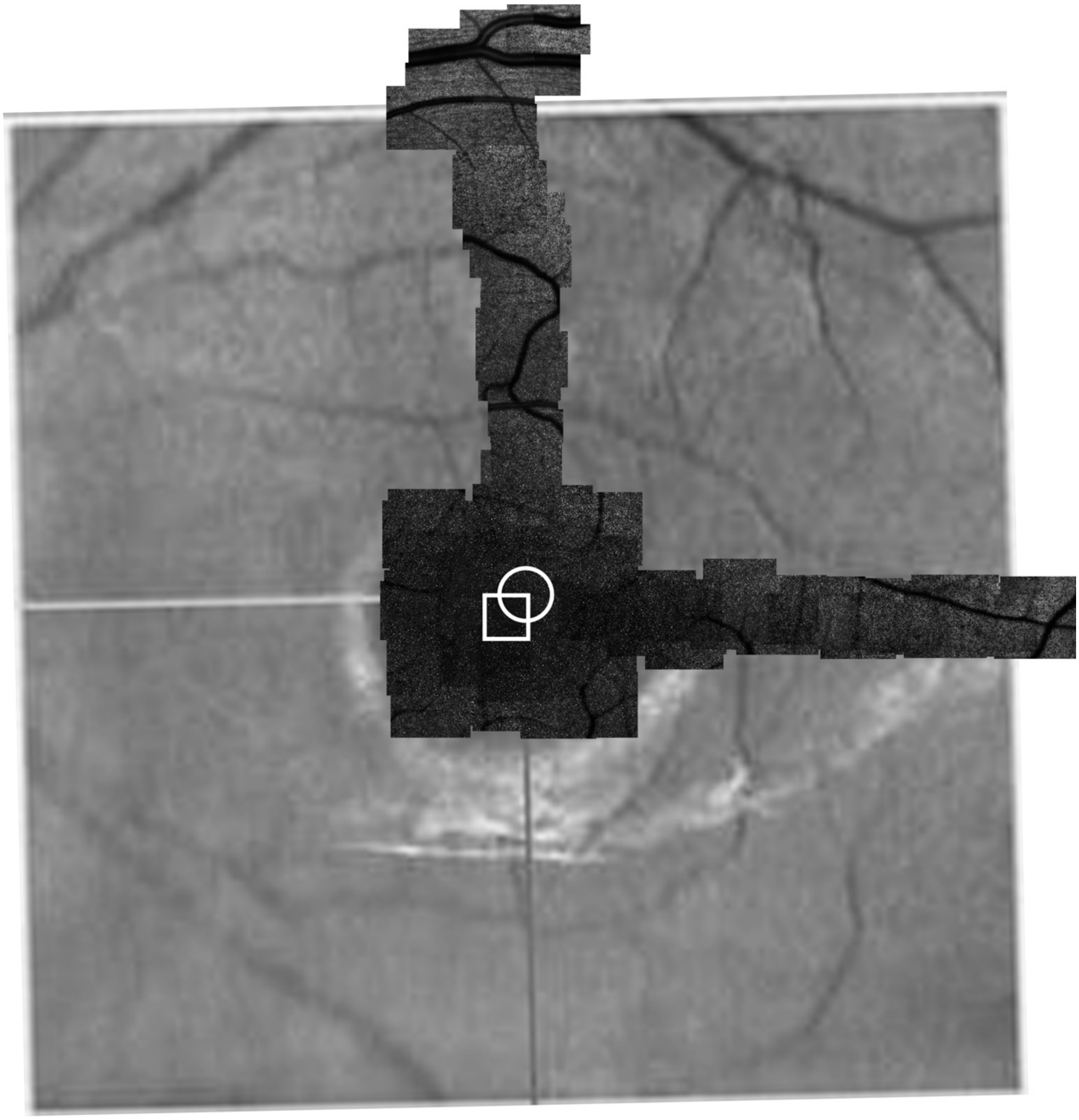

Supplemental Figure 3

Confocal (left) and corresponding split-detection images (right), centered on the location of peak cell density, of all ten patients with blue cone monochromacy for whom both modalities were analyzable. The anatomical fovea is marked with a star for all patients except JC_10558, for whom alignment between modalities was not possible.

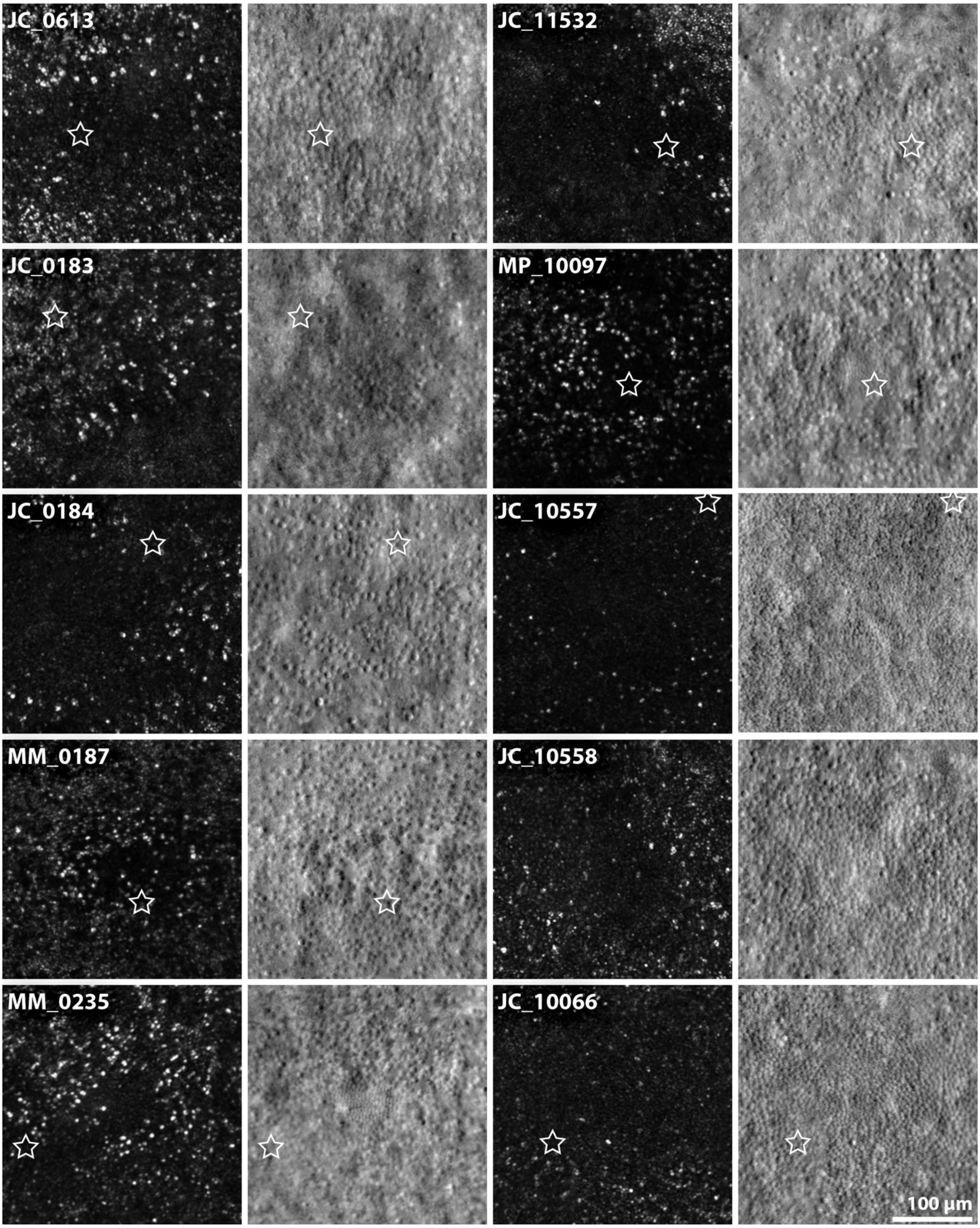
JC_0613
JC_11532
JC_0183
MP_10097
JC_0184
JC_10557
MM_0187
JC_10558
MM_0235
JC_10066
100 µm
